# Death of Dr. G. M. Scarlett

**Published:** 1856-11

**Authors:** 


### DEATH OF DR. G. M. SCARLETT.

It is with deep regret that we have to record the death of Dr. G. M. Scarlett, one of the graduates of the first session of the Atlanta Medical College; he died during the month of September, at the residence of his father, Glynn Co., Ga. We had the melancholy pleasure of seeing him a few days before his death, and shall not soon forget the gratification which our visit seemed to afford him; and the calmness and even cheerfullness with which he bore the distressing disease under which he labored.

We have rarely met with a more singular case of disease than we saw exhibited in the person of this interesting young gentleman.

After graduating here, he went to Savannah last winter with a view of prosecuting his medical studies, and while in attendance upon the lectures in the Savannah Medical College, he contracted (as we were informed) Pneumonia, which was followed by suppuration of the left lung—the abscess pointing and discharging externally through the parietes of the chest; the process of suppuration continued, and when we saw him, the pus was discharging from a second opening through the walls of the chest (the first having closed) the lung being almost entirely destroyed; some six or eight months having elapsed since the acute attack.

This is the first death among our Alumni that has come to our knowledge, and we hope that it may be long before it shall be our painful task to record the death of another.

